# 1-Methyl-4-[(1*E*,3*E*)-4-phenyl­buta-1,3-dien­yl]pyridinium iodide monohydrate^1^

**DOI:** 10.1107/S1600536810006045

**Published:** 2010-02-20

**Authors:** Hoong-Kun Fun, Kullapa Chanawanno, Chanasuk Surasit, Suchada Chantrapromma

**Affiliations:** a X-ray Crystallography Unit, School of Physics, Universiti Sains Malaysia, 11800 USM, Penang, Malaysia; b Crystal Materials Research Unit, Department of Chemistry, Faculty of Science, Prince of Songkla University, Hat-Yai, Songkhla 90112, Thailand

## Abstract

The asymmetric unit of the title compound, C_16_H_16_N^+^·I^−^·H_2_O, contains two 1-methyl-4-{[(1*E*,3*E*)-4-phenyl­buta-1,3-dien­yl]}pyridinium cations, two iodide ions and two solvent water mol­ecules. The cation is twisted slightly, the dihedral angle between the pyridinium and the phenyl rings being 10.68 (18)° in one mol­ecule and 18.9 (3)° in the other. The two water mol­ecules are disordered over three positions with site-occupancy ratio of 0.9/0.7/0.4. In the crystal packing, the cations are arranged into ribbons along the *b* axis with the iodide ions and water mol­ecules located between adjacent cations. The cations are linked to the iodide ions and water mol­ecules by weak C—H⋯I and C—H⋯O inter­actions, respectively. These inter­actions together with O—H⋯I hydrogen bonds link the mol­ecules into a two-dimensional network parallel to the *bc *plane. π⋯π inter­actions with a centroid–centroid distance of 3.669 (2) Å are also observed.

## Related literature

For bond-length data, see: Allen *et al.* (1987 ▶). For background to non-linear optical materials research, see: Raimundo *et al.* (2002 ▶). For related structures, see: Chantrapromma *et al.* (2009*a* ▶,*b* ▶), Fun *et al.* (2009 ▶). For the stability of the temperature controller used in the data collection, see: Cosier & Glazer, (1986 ▶).
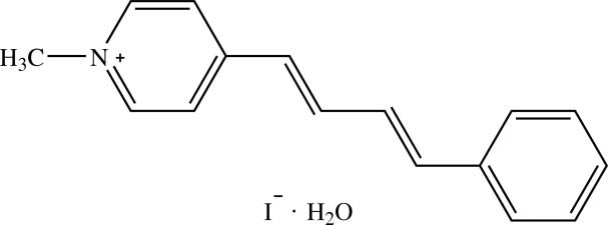


## Experimental

### 

#### Crystal data

C_16_H_16_N^+^·I^−^·H_2_O
*M* *_r_* = 367.21
Monoclinic, 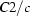

*a* = 32.5600 (6) Å
*b* = 12.6414 (2) Å
*c* = 16.5602 (3) Å
β = 111.180 (1)°
*V* = 6355.81 (19) Å^3^
*Z* = 16
Mo *K*α radiation
μ = 2.01 mm^−1^
*T* = 100 K
0.55 × 0.20 × 0.20 mm

#### Data collection

Bruker APEXII CCD area detector diffractometer
Absorption correction: multi-scan (*SADABS*; Bruker, 2005 ▶) *T*_min_ = 0.407, *T*_max_ = 0.694
36570 measured reflections
9279 independent reflections
6818 reflections with *I* > 2σ(*I*)
*R*_int_ = 0.031

#### Refinement

*R*[*F* ^2^ > 2σ(*F* ^2^)] = 0.046
*wR*(*F* ^2^) = 0.117
*S* = 1.02
9279 reflections
357 parameters
H-atom parameters constrained
Δρ_max_ = 2.40 e Å^−3^
Δρ_min_ = −1.87 e Å^−3^

### 

Data collection: *APEX2* (Bruker, 2005 ▶); cell refinement: *SAINT* (Bruker, 2005 ▶); data reduction: *SAINT*; program(s) used to solve structure: *SHELXTL* (Sheldrick, 2008 ▶); program(s) used to refine structure: *SHELXTL*; molecular graphics: *SHELXTL*; software used to prepare material for publication: *SHELXTL* and *PLATON* (Spek, 2009 ▶).

## Supplementary Material

Crystal structure: contains datablocks global, I. DOI: 10.1107/S1600536810006045/sj2731sup1.cif

Structure factors: contains datablocks I. DOI: 10.1107/S1600536810006045/sj2731Isup2.hkl

Additional supplementary materials:  crystallographic information; 3D view; checkCIF report

## Figures and Tables

**Table 1:** Hydrogen-bond geometry (Å, °)

*D*—H⋯*A*	*D*—H	H⋯*A*	*D*⋯*A*	*D*—H⋯*A*
O2*W*—H2*W*2⋯I1*A*^i^	0.79	2.88	3.655 (8)	166
C3*B*—H3*B*⋯O1*W*^ii^	0.93	2.51	3.399 (8)	161
C16*A*—H16*A*⋯I1*A*^iii^	0.96	3.05	3.992 (4)	167

Symmetry codes: (i) 
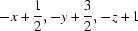
; (ii) 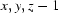; (iii) 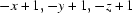.

**Fig. 1.**
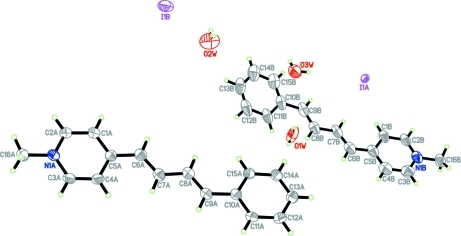
The asymmetric unit of the title compound, with 50% probability displacement ellipsoids and the atom-numbering scheme.

**Fig. 2.**
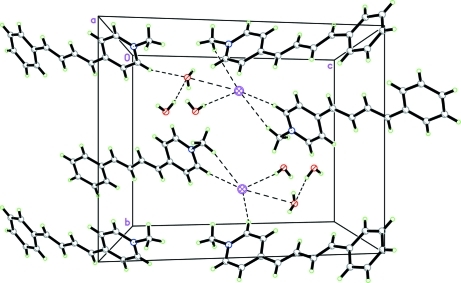
The crystal packing of the title compound viewed down the a axis. Hydrogen bonds, weak C—H···O and C—H···I interactions are shown as dashed lines.

## supplementary crystallographic information

### Comment

It was known that the non-linear optic (NLO) materials require molecular first hyperpolarizability (*β*) and at the molecular level, compounds likely to exhibit large *β* values must have polarizable electrons (i.e. π-electrons) spread over a large distance. Thus, organic dipolar compounds with extended π systems having terminal donor and acceptor groups are likely to exhibit large π values (Raimundo *et al.*, 2002). We have previous reported the crystal structures of the NLO-active compounds (Chantrapromma *et al.*, 2009*a*, *b*; Fun *et al.*, 2009) in which the cations consist of an ethenyl bridge between two rings. The title compound was designed and synthesized by extending the π-conjugate systems of the cation with an expectation for better NLO properties. However, the title compound crystallizes in centrosymmetric *C*2/*c* space group and does not exhibit second-order nonlinear optical properties.

The asymmetric unit of the title compound, C_16_H_16_N^+^.I^-^.H_2_O, Fig 1, comprises two 1-methyl-4-{[(1*E*,3*E*)-4-phenylbuta-1,3-dienyl]}pyridinium cations, two iodide ions and two solvent water molecules. The cation is slightly twisted with the dihedral angle between the pyridinium and phenyl rings being 10.68 (18) ° in molecule *A* [18.9 (3)° in molecule *B*]. The buta-1,3-dienyl moiety (C6–C9) is almost planar with the r.m.s of 0.0046 (5) Å in molecule *A* [0.0283 (5) Å in molecule *B*] and the torsion angles C6–C7–C8–C9 = 179.1 (4)° in molecule *A* [174.2 (4)° in molecule *B*]. This unit makes the dihedral angles of 6.4 (4) and 5.4 (4)° with the pyridinium and phenyl ring, respectively in molecule *A* [the corresponding values are 5.7 (5) and 13.4 (5)° in molecule *B*]. The two water molecules are disordered over three positions with the site-occupancy ratio of 0.9/0.7/0.4. The bond lengths of cations are in normal ranges (Allen *et al.*, 1987) and comparable to those in related structures (Chantrapromma *et al.*, 2009*a*, *b*, Fun *et al.*, 2009).

In the crystal packing (Fig. 2), the cations are arranged into ribbons along the *b* axis with the iodide ions and water molecules located between adjacent cations. The cations are linked to the iodide ions and water molecules by C—H···I and C—H···O weak interactions (Table 1), respectively whereas water molecules form O—H···I hydrogen bonds (Table 1) with iodide ions. These interactions linked the molecules into two-dimensional networks parallel to the *bc* plane. π···π interactions involving pyridinium and phenyl rings was also observed with the distance of Cg_1_···Cg_2_ = 3.669 (2) Å (symmetry code: 3/2-x, 1/2-y, -z); Cg_1_ and Cg_2_ are the centroids of N1A/C1A–C5A and C10A–C15A rings, respectively.

### Experimental

The title compound was prepared by mixing 1:1:1 molar ratio solutions of 1,4-dimethylpyridinium iodide (2 g, 8.5 mmol), cinnamaldehyde (1.1 g, 8.5 mmol) and piperidine (0.84 ml, 8.5 mmol) in methanol (40 ml). The resulting solution was refluxed for 3 h under a nitrogen atmosphere. The yellow solid which formed was filtered, washed with diethylether and recrystallized from methanol by slow evaporation at room temperature to yield the yellow block-shaped single crystals suitable for *x*-ray diffraction analysis over a few weeks (Mp. 496-498 K).

### Refinement

All H atoms were positioned geometrically and allowed to ride on their parent atoms, with d(O-H) = 0.71-0.92 Å, d(C-H) = 0.93 Å for aromatic and CH and 0.96 Å for CH_3_ atoms. The *U*_iso_ values were constrained to be 1.5*U*_eq_ of the carrier atom for methyl H atoms and 1.2*U*_eq_ for the remaining H atoms. A rotating group model was used for the methyl groups. The two water molecules are disordered over three sites with occupancies 0.931 (9), 0.695 (9) and 0.354 (9), respectively. In the final refinement, this ratio was fixed as 0.90 : 0.70 : 0.40. The highest residual electron density peak is located at 0.84 Å from I1B and the deepest hole is located at 0.83 Å from I1B.

### Crystal data

**Table:**

C_16_H_16_N^+^·I^−^·H_2_O	*F*(000) = 2912
*M**_r_* = 367.21	*D*_x_ = 1.535 Mg m^−^^3^
Monoclinic, *C*2/*c*	Melting point = 496–498 K
Hall symbol: -C 2yc	Mo *K*α radiation, λ = 0.71073 Å
*a* = 32.5600 (6) Å	Cell parameters from 9279 reflections
*b* = 12.6414 (2) Å	θ = 1.7–30.0°
*c* = 16.5602 (3) Å	µ = 2.01 mm^−^^1^
β = 111.180 (1)°	*T* = 100 K
*V* = 6355.81 (19) Å^3^	Block, yellow
*Z* = 16	0.55 × 0.20 × 0.20 mm

### Data collection

**Table:**

Bruker APEXII CCD area detector diffractometer	9279 independent reflections
Radiation source: sealed tube	6818 reflections with *I* > 2σ(*I*)
graphite	*R*_int_ = 0.031
φ and ω scans	θ_max_ = 30.0°, θ_min_ = 1.7°
Absorption correction: multi-scan (*SADABS*; Bruker, 2005)	*h* = −39→45
*T*_min_ = 0.407, *T*_max_ = 0.694	*k* = −17→17
36570 measured reflections	*l* = −23→23

### Refinement

**Table:**

Refinement on *F*^2^	Primary atom site location: structure-invariant direct methods
Least-squares matrix: full	Secondary atom site location: difference Fourier map
*R*[*F*^2^ > 2σ(*F*^2^)] = 0.046	Hydrogen site location: inferred from neighbouring sites
*wR*(*F*^2^) = 0.117	H-atom parameters constrained
*S* = 1.01	*w* = 1/[σ^2^(*F*_o_^2^) + (0.0415*P*)^2^ + 36.1246*P*] where *P* = (*F*_o_^2^ + 2*F*_c_^2^)/3
9279 reflections	(Δ/σ)_max_ = 0.003
357 parameters	Δρ_max_ = 2.40 e Å^−^^3^
0 restraints	Δρ_min_ = −1.87 e Å^−^^3^

### Special details

**Table:**

Experimental. The crystal was placed in the cold stream of an Oxford Cryosystems Cobra open-flow nitrogen cryostat (Cosier & Glazer, 1986) operating at 100.0 (1) K.
Geometry. All esds (except the esd in the dihedral angle between two l.s. planes) are estimated using the full covariance matrix. The cell esds are taken into account individually in the estimation of esds in distances, angles and torsion angles; correlations between esds in cell parameters are only used when they are defined by crystal symmetry. An approximate (isotropic) treatment of cell esds is used for estimating esds involving l.s. planes.
Refinement. Refinement of F^2^ against ALL reflections. The weighted R-factor wR and goodness of fit S are based on F^2^, conventional R-factors R are based on F, with F set to zero for negative F^2^. The threshold expression of F^2^ > 2sigma(F^2^) is used only for calculating R-factors(gt) etc. and is not relevant to the choice of reflections for refinement. R-factors based on F^2^ are statistically about twice as large as those based on F, and R- factors based on ALL data will be even larger.

### Fractional atomic coordinates and isotropic or equivalent isotropic displacement parameters (Å^2^)

**Table:**

	*x*	*y*	*z*	*U*_iso_*/*U*_eq_	Occ. (<1)
I1A	0.006481 (8)	0.74812 (2)	0.492960 (16)	0.03922 (8)	
I1B	0.825988 (11)	0.71635 (2)	0.18855 (3)	0.06328 (12)	
N1A	0.90440 (10)	0.0474 (2)	0.29244 (19)	0.0305 (6)	
C1A	0.84641 (12)	0.1693 (3)	0.2688 (2)	0.0302 (7)	
H1A	0.8356	0.2302	0.2856	0.036*	
C2A	0.88809 (12)	0.1352 (3)	0.3159 (2)	0.0303 (7)	
H2A	0.9053	0.1729	0.3646	0.036*	
C3A	0.87910 (13)	−0.0101 (3)	0.2239 (2)	0.0368 (8)	
H3A	0.8904	−0.0719	0.2094	0.044*	
C4A	0.83732 (13)	0.0206 (3)	0.1755 (2)	0.0360 (8)	
H4A	0.8204	−0.0206	0.1289	0.043*	
C5A	0.81968 (12)	0.1135 (3)	0.1953 (2)	0.0303 (7)	
C6A	0.77644 (12)	0.1532 (3)	0.1426 (2)	0.0348 (8)	
H6A	0.7662	0.2132	0.1619	0.042*	
C7A	0.75006 (13)	0.1094 (3)	0.0677 (3)	0.0378 (8)	
H7A	0.7597	0.0476	0.0497	0.045*	
C8A	0.70796 (12)	0.1511 (3)	0.0135 (2)	0.0358 (8)	
H8A	0.6976	0.2122	0.0310	0.043*	
C9A	0.68303 (12)	0.1052 (3)	−0.0615 (2)	0.0340 (7)	
H9A	0.6943	0.0438	−0.0765	0.041*	
C10A	0.64019 (12)	0.1407 (3)	−0.1220 (2)	0.0311 (7)	
C11A	0.62086 (12)	0.0866 (3)	−0.2003 (2)	0.0328 (7)	
H11A	0.6352	0.0286	−0.2125	0.039*	
C12A	0.58082 (12)	0.1182 (3)	−0.2597 (3)	0.0367 (8)	
H12A	0.5686	0.0817	−0.3117	0.044*	
C13A	0.55865 (12)	0.2036 (3)	−0.2427 (3)	0.0361 (8)	
H13A	0.5318	0.2254	−0.2832	0.043*	
C14A	0.57699 (12)	0.2566 (3)	−0.1641 (2)	0.0338 (7)	
H14A	0.5618	0.3126	−0.1513	0.041*	
C15A	0.61757 (12)	0.2269 (3)	−0.1047 (2)	0.0320 (7)	
H15A	0.6299	0.2642	−0.0532	0.038*	
C16A	0.95039 (13)	0.0138 (3)	0.3392 (3)	0.0380 (8)	
H16A	0.9642	0.0618	0.3861	0.057*	
H16B	0.9662	0.0141	0.3002	0.057*	
H16C	0.9506	−0.0563	0.3617	0.057*	
N1B	0.07132 (13)	0.4780 (3)	−0.0525 (3)	0.0509 (9)	
C1B	0.12480 (15)	0.5134 (4)	0.0860 (3)	0.0503 (11)	
H1B	0.1340	0.5532	0.1366	0.060*	
C2B	0.08526 (15)	0.5343 (4)	0.0221 (3)	0.0490 (11)	
H2B	0.0676	0.5881	0.0300	0.059*	
C3B	0.09613 (17)	0.4012 (4)	−0.0651 (4)	0.0586 (12)	
H3B	0.0864	0.3634	−0.1168	0.070*	
C4B	0.13613 (17)	0.3770 (4)	−0.0025 (4)	0.0595 (13)	
H4B	0.1531	0.3229	−0.0125	0.071*	
C5B	0.15160 (14)	0.4319 (4)	0.0753 (3)	0.0496 (11)	
C6B	0.19467 (14)	0.4065 (4)	0.1392 (3)	0.0517 (11)	
H6B	0.2101	0.3507	0.1270	0.062*	
C7B	0.21354 (13)	0.4573 (4)	0.2139 (3)	0.0488 (11)	
H7B	0.1972	0.5097	0.2280	0.059*	
C8B	0.25747 (14)	0.4369 (4)	0.2747 (3)	0.0491 (11)	
H8B	0.2734	0.3806	0.2646	0.059*	
C9B	0.27587 (14)	0.4970 (4)	0.3451 (3)	0.0509 (12)	
H9B	0.2578	0.5473	0.3563	0.061*	
C10B	0.32147 (15)	0.4919 (4)	0.4064 (3)	0.0519 (12)	
C11B	0.35014 (14)	0.4109 (4)	0.4035 (3)	0.0550 (13)	
H11B	0.3402	0.3554	0.3644	0.066*	
C12B	0.39410 (16)	0.4145 (5)	0.4602 (4)	0.0646 (16)	
H12B	0.4134	0.3607	0.4593	0.077*	
C13B	0.40872 (18)	0.4975 (5)	0.5174 (4)	0.0699 (17)	
H13B	0.4381	0.4997	0.5539	0.084*	
C14B	0.3806 (2)	0.5774 (5)	0.5217 (3)	0.0703 (16)	
H14B	0.3908	0.6328	0.5609	0.084*	
C15B	0.33693 (17)	0.5734 (5)	0.4665 (3)	0.0601 (13)	
H15B	0.3176	0.6260	0.4697	0.072*	
C16B	0.02813 (16)	0.5012 (4)	−0.1198 (3)	0.0622 (14)	
H16D	0.0291	0.4851	−0.1758	0.093*	
H16E	0.0213	0.5748	−0.1175	0.093*	
H16F	0.0059	0.4589	−0.1100	0.093*	
O1W	0.05902 (16)	0.3235 (5)	0.7242 (3)	0.104 (2)	0.90
H1W1	0.0640	0.2698	0.7188	0.156*	0.90
H2W1	0.0761	0.3618	0.7007	0.156*	0.90
O2W	0.4677 (3)	0.6439 (6)	0.6879 (4)	0.103 (2)	0.70
H1W2	0.4610	0.6930	0.7206	0.154*	0.70
H2W2	0.4770	0.6733	0.6557	0.154*	0.70
O3W	0.2541 (4)	0.6530 (9)	0.5171 (7)	0.087 (3)	0.40
H1W3	0.2374	0.6726	0.4678	0.130*	0.40
H2W3	0.2766	0.6895	0.5289	0.130*	0.40

### Atomic displacement parameters (Å^2^)

**Table:**

	*U*^11^	*U*^22^	*U*^33^	*U*^12^	*U*^13^	*U*^23^
I1A	0.03478 (13)	0.03914 (14)	0.03595 (13)	0.00447 (10)	0.00338 (9)	−0.00831 (10)
I1B	0.04728 (17)	0.03069 (15)	0.0990 (3)	0.00016 (12)	0.01098 (17)	−0.00940 (15)
N1A	0.0356 (15)	0.0254 (14)	0.0325 (15)	0.0005 (11)	0.0145 (13)	0.0036 (11)
C1A	0.0348 (18)	0.0286 (17)	0.0318 (17)	−0.0008 (13)	0.0177 (14)	−0.0004 (13)
C2A	0.0353 (18)	0.0287 (17)	0.0301 (17)	−0.0033 (13)	0.0157 (14)	−0.0020 (13)
C3A	0.047 (2)	0.0248 (17)	0.0371 (19)	0.0014 (15)	0.0132 (17)	−0.0033 (14)
C4A	0.044 (2)	0.0291 (18)	0.0315 (18)	−0.0033 (15)	0.0096 (16)	−0.0054 (14)
C5A	0.0333 (17)	0.0289 (17)	0.0303 (16)	−0.0024 (13)	0.0134 (14)	0.0044 (13)
C6A	0.0359 (19)	0.0325 (18)	0.0377 (19)	−0.0001 (14)	0.0152 (16)	0.0025 (14)
C7A	0.039 (2)	0.0339 (19)	0.041 (2)	0.0024 (15)	0.0151 (17)	0.0042 (15)
C8A	0.0372 (19)	0.0327 (18)	0.0389 (19)	0.0021 (15)	0.0154 (16)	0.0020 (15)
C9A	0.0367 (19)	0.0281 (17)	0.0390 (19)	0.0029 (14)	0.0160 (16)	0.0028 (14)
C10A	0.0334 (17)	0.0278 (17)	0.0329 (17)	0.0009 (13)	0.0131 (14)	0.0032 (13)
C11A	0.0372 (18)	0.0230 (16)	0.0405 (19)	−0.0008 (13)	0.0170 (16)	−0.0024 (13)
C12A	0.0357 (19)	0.0347 (19)	0.040 (2)	−0.0062 (15)	0.0138 (16)	−0.0064 (15)
C13A	0.0297 (17)	0.037 (2)	0.042 (2)	−0.0003 (14)	0.0136 (15)	0.0001 (15)
C14A	0.0335 (17)	0.0332 (18)	0.0379 (18)	0.0049 (14)	0.0166 (15)	−0.0010 (15)
C15A	0.0348 (18)	0.0308 (18)	0.0323 (17)	0.0005 (13)	0.0143 (15)	−0.0030 (13)
C16A	0.040 (2)	0.0333 (19)	0.038 (2)	0.0058 (15)	0.0110 (16)	0.0007 (15)
N1B	0.046 (2)	0.044 (2)	0.062 (2)	−0.0065 (16)	0.0187 (19)	0.0218 (18)
C1B	0.046 (2)	0.048 (3)	0.057 (3)	0.0014 (19)	0.019 (2)	0.020 (2)
C2B	0.046 (2)	0.044 (2)	0.061 (3)	0.0042 (19)	0.025 (2)	0.022 (2)
C3B	0.056 (3)	0.053 (3)	0.070 (3)	−0.011 (2)	0.027 (3)	0.002 (2)
C4B	0.050 (3)	0.055 (3)	0.082 (4)	−0.001 (2)	0.033 (3)	0.007 (3)
C5B	0.040 (2)	0.045 (2)	0.069 (3)	−0.0013 (18)	0.025 (2)	0.020 (2)
C6B	0.039 (2)	0.045 (2)	0.077 (3)	0.0034 (18)	0.027 (2)	0.017 (2)
C7B	0.034 (2)	0.047 (2)	0.073 (3)	0.0072 (18)	0.028 (2)	0.026 (2)
C8B	0.038 (2)	0.049 (3)	0.068 (3)	0.0052 (18)	0.027 (2)	0.024 (2)
C9B	0.037 (2)	0.060 (3)	0.066 (3)	0.0106 (19)	0.031 (2)	0.026 (2)
C10B	0.038 (2)	0.067 (3)	0.057 (3)	0.006 (2)	0.025 (2)	0.031 (2)
C11B	0.041 (2)	0.064 (3)	0.066 (3)	0.009 (2)	0.026 (2)	0.037 (2)
C12B	0.045 (3)	0.074 (4)	0.083 (4)	0.011 (2)	0.032 (3)	0.050 (3)
C13B	0.050 (3)	0.096 (5)	0.059 (3)	−0.005 (3)	0.013 (2)	0.043 (3)
C14B	0.071 (4)	0.097 (5)	0.047 (3)	0.002 (3)	0.026 (3)	0.024 (3)
C15B	0.057 (3)	0.083 (4)	0.051 (3)	0.008 (3)	0.033 (2)	0.021 (3)
C16B	0.052 (3)	0.059 (3)	0.065 (3)	−0.009 (2)	0.009 (2)	0.027 (2)
O1W	0.062 (3)	0.163 (6)	0.068 (3)	0.025 (3)	0.000 (2)	−0.037 (3)
O2W	0.143 (7)	0.107 (5)	0.084 (4)	−0.027 (5)	0.073 (5)	−0.010 (4)
O3W	0.109 (9)	0.064 (6)	0.071 (7)	0.016 (6)	0.014 (6)	−0.001 (5)

### Geometric parameters (Å, °)

**Table:**

N1A—C2A	1.346 (4)	C1B—C2B	1.364 (6)
N1A—C3A	1.349 (5)	C1B—C5B	1.402 (7)
N1A—C16A	1.478 (5)	C1B—H1B	0.9300
C1A—C2A	1.368 (5)	C2B—H2B	0.9300
C1A—C5A	1.403 (5)	C3B—C4B	1.374 (7)
C1A—H1A	0.9300	C3B—H3B	0.9300
C2A—H2A	0.9300	C4B—C5B	1.388 (7)
C3A—C4A	1.362 (5)	C4B—H4B	0.9300
C3A—H3A	0.9300	C5B—C6B	1.456 (6)
C4A—C5A	1.397 (5)	C6B—C7B	1.330 (7)
C4A—H4A	0.9300	C6B—H6B	0.9300
C5A—C6A	1.452 (5)	C7B—C8B	1.444 (6)
C6A—C7A	1.347 (5)	C7B—H7B	0.9300
C6A—H6A	0.9300	C8B—C9B	1.338 (7)
C7A—C8A	1.440 (5)	C8B—H8B	0.9300
C7A—H7A	0.9300	C9B—C10B	1.466 (6)
C8A—C9A	1.345 (5)	C9B—H9B	0.9300
C8A—H8A	0.9300	C10B—C15B	1.394 (8)
C9A—C10A	1.464 (5)	C10B—C11B	1.399 (7)
C9A—H9A	0.9300	C11B—C12B	1.401 (7)
C10A—C11A	1.398 (5)	C11B—H11B	0.9300
C10A—C15A	1.401 (5)	C12B—C13B	1.378 (9)
C11A—C12A	1.378 (5)	C12B—H12B	0.9300
C11A—H11A	0.9300	C13B—C14B	1.382 (9)
C12A—C13A	1.384 (5)	C13B—H13B	0.9300
C12A—H12A	0.9300	C14B—C15B	1.387 (8)
C13A—C14A	1.392 (5)	C14B—H14B	0.9300
C13A—H13A	0.9300	C15B—H15B	0.9300
C14A—C15A	1.384 (5)	C16B—H16D	0.9600
C14A—H14A	0.9300	C16B—H16E	0.9600
C15A—H15A	0.9300	C16B—H16F	0.9600
C16A—H16A	0.9600	O1W—H1W1	0.7106
C16A—H16B	0.9600	O1W—H2W1	0.9232
C16A—H16C	0.9600	O2W—H1W2	0.8994
N1B—C3B	1.326 (6)	O2W—H2W2	0.7950
N1B—C2B	1.355 (6)	O3W—H1W3	0.8376
N1B—C16B	1.474 (6)	O3W—H2W3	0.8268
			
C2A—N1A—C3A	120.2 (3)	C2B—N1B—C16B	119.9 (4)
C2A—N1A—C16A	121.1 (3)	C2B—C1B—C5B	120.0 (5)
C3A—N1A—C16A	118.7 (3)	C2B—C1B—H1B	120.0
C2A—C1A—C5A	120.9 (3)	C5B—C1B—H1B	120.0
C2A—C1A—H1A	119.6	N1B—C2B—C1B	121.1 (5)
C5A—C1A—H1A	119.6	N1B—C2B—H2B	119.5
N1A—C2A—C1A	120.5 (3)	C1B—C2B—H2B	119.5
N1A—C2A—H2A	119.7	N1B—C3B—C4B	120.7 (5)
C1A—C2A—H2A	119.7	N1B—C3B—H3B	119.7
N1A—C3A—C4A	121.1 (3)	C4B—C3B—H3B	119.7
N1A—C3A—H3A	119.4	C3B—C4B—C5B	121.0 (5)
C4A—C3A—H3A	119.4	C3B—C4B—H4B	119.5
C3A—C4A—C5A	120.6 (3)	C5B—C4B—H4B	119.5
C3A—C4A—H4A	119.7	C4B—C5B—C1B	116.8 (4)
C5A—C4A—H4A	119.7	C4B—C5B—C6B	119.7 (5)
C4A—C5A—C1A	116.5 (3)	C1B—C5B—C6B	123.4 (5)
C4A—C5A—C6A	122.8 (3)	C7B—C6B—C5B	124.7 (5)
C1A—C5A—C6A	120.7 (3)	C7B—C6B—H6B	117.6
C7A—C6A—C5A	124.6 (4)	C5B—C6B—H6B	117.6
C7A—C6A—H6A	117.7	C6B—C7B—C8B	124.8 (5)
C5A—C6A—H6A	117.7	C6B—C7B—H7B	117.6
C6A—C7A—C8A	124.7 (4)	C8B—C7B—H7B	117.6
C6A—C7A—H7A	117.6	C9B—C8B—C7B	121.7 (5)
C8A—C7A—H7A	117.6	C9B—C8B—H8B	119.1
C9A—C8A—C7A	122.6 (4)	C7B—C8B—H8B	119.1
C9A—C8A—H8A	118.7	C8B—C9B—C10B	127.0 (5)
C7A—C8A—H8A	118.7	C8B—C9B—H9B	116.5
C8A—C9A—C10A	127.4 (3)	C10B—C9B—H9B	116.5
C8A—C9A—H9A	116.3	C15B—C10B—C11B	119.5 (5)
C10A—C9A—H9A	116.3	C15B—C10B—C9B	118.5 (5)
C11A—C10A—C15A	118.4 (3)	C11B—C10B—C9B	122.0 (5)
C11A—C10A—C9A	118.9 (3)	C10B—C11B—C12B	119.1 (6)
C15A—C10A—C9A	122.7 (3)	C10B—C11B—H11B	120.5
C12A—C11A—C10A	120.9 (3)	C12B—C11B—H11B	120.5
C12A—C11A—H11A	119.6	C13B—C12B—C11B	120.1 (5)
C10A—C11A—H11A	119.6	C13B—C12B—H12B	120.0
C11A—C12A—C13A	120.6 (4)	C11B—C12B—H12B	120.0
C11A—C12A—H12A	119.7	C12B—C13B—C14B	121.4 (5)
C13A—C12A—H12A	119.7	C12B—C13B—H13B	119.3
C12A—C13A—C14A	119.2 (4)	C14B—C13B—H13B	119.3
C12A—C13A—H13A	120.4	C13B—C14B—C15B	118.7 (6)
C14A—C13A—H13A	120.4	C13B—C14B—H14B	120.7
C15A—C14A—C13A	120.7 (3)	C15B—C14B—H14B	120.7
C15A—C14A—H14A	119.7	C14B—C15B—C10B	121.2 (5)
C13A—C14A—H14A	119.7	C14B—C15B—H15B	119.4
C14A—C15A—C10A	120.2 (3)	C10B—C15B—H15B	119.4
C14A—C15A—H15A	119.9	N1B—C16B—H16D	109.5
C10A—C15A—H15A	119.9	N1B—C16B—H16E	109.5
N1A—C16A—H16A	109.5	H16D—C16B—H16E	109.5
N1A—C16A—H16B	109.5	N1B—C16B—H16F	109.5
H16A—C16A—H16B	109.5	H16D—C16B—H16F	109.5
N1A—C16A—H16C	109.5	H16E—C16B—H16F	109.5
H16A—C16A—H16C	109.5	H1W1—O1W—H2W1	104.4
H16B—C16A—H16C	109.5	H1W2—O2W—H2W2	108.4
C3B—N1B—C2B	120.4 (4)	H1W3—O3W—H2W3	105.9
C3B—N1B—C16B	119.7 (5)		
			
C3A—N1A—C2A—C1A	−2.6 (5)	C3B—N1B—C2B—C1B	−0.3 (6)
C16A—N1A—C2A—C1A	175.8 (3)	C16B—N1B—C2B—C1B	179.0 (4)
C5A—C1A—C2A—N1A	0.3 (5)	C5B—C1B—C2B—N1B	−0.5 (6)
C2A—N1A—C3A—C4A	2.1 (5)	C2B—N1B—C3B—C4B	0.5 (7)
C16A—N1A—C3A—C4A	−176.2 (4)	C16B—N1B—C3B—C4B	−178.8 (4)
N1A—C3A—C4A—C5A	0.6 (6)	N1B—C3B—C4B—C5B	0.0 (7)
C3A—C4A—C5A—C1A	−2.7 (5)	C3B—C4B—C5B—C1B	−0.8 (7)
C3A—C4A—C5A—C6A	176.0 (4)	C3B—C4B—C5B—C6B	−178.2 (4)
C2A—C1A—C5A—C4A	2.3 (5)	C2B—C1B—C5B—C4B	1.0 (6)
C2A—C1A—C5A—C6A	−176.4 (3)	C2B—C1B—C5B—C6B	178.3 (4)
C4A—C5A—C6A—C7A	−3.9 (6)	C4B—C5B—C6B—C7B	176.7 (4)
C1A—C5A—C6A—C7A	174.8 (4)	C1B—C5B—C6B—C7B	−0.6 (7)
C5A—C6A—C7A—C8A	−177.3 (3)	C5B—C6B—C7B—C8B	−175.5 (4)
C6A—C7A—C8A—C9A	179.1 (4)	C6B—C7B—C8B—C9B	174.2 (4)
C7A—C8A—C9A—C10A	−179.3 (4)	C7B—C8B—C9B—C10B	−173.0 (4)
C8A—C9A—C10A—C11A	175.0 (4)	C8B—C9B—C10B—C15B	168.2 (4)
C8A—C9A—C10A—C15A	−5.1 (6)	C8B—C9B—C10B—C11B	−8.8 (7)
C15A—C10A—C11A—C12A	1.0 (5)	C15B—C10B—C11B—C12B	−1.1 (6)
C9A—C10A—C11A—C12A	−179.2 (3)	C9B—C10B—C11B—C12B	175.8 (4)
C10A—C11A—C12A—C13A	−0.7 (6)	C10B—C11B—C12B—C13B	−0.6 (6)
C11A—C12A—C13A—C14A	−0.9 (6)	C11B—C12B—C13B—C14B	1.4 (7)
C12A—C13A—C14A—C15A	2.2 (6)	C12B—C13B—C14B—C15B	−0.4 (7)
C13A—C14A—C15A—C10A	−1.9 (6)	C13B—C14B—C15B—C10B	−1.3 (7)
C11A—C10A—C15A—C14A	0.3 (5)	C11B—C10B—C15B—C14B	2.1 (6)
C9A—C10A—C15A—C14A	−179.5 (3)	C9B—C10B—C15B—C14B	−175.0 (4)

### Hydrogen-bond geometry (Å, °)

**Table:**

*D*—H···*A*	*D*—H	H···*A*	*D*···*A*	*D*—H···*A*
O2W—H2W2···I1A^i^	0.79	2.88	3.655 (8)	166
C3B—H3B···O1W^ii^	0.93	2.51	3.399 (8)	161
C16A—H16A···I1A^iii^	0.96	3.05	3.992 (4)	167

Symmetry codes: (i) −*x*+1/2, −*y*+3/2, −*z*+1; (ii) *x*, *y*, *z*−1; (iii) −*x*+1, −*y*+1, −*z*+1.
